# Depressive symptoms in HIV-infected and seronegative control subjects in Cameroon: Effect of age, education and gender

**DOI:** 10.1371/journal.pone.0171956

**Published:** 2017-02-23

**Authors:** Georgette D. Kanmogne, Fang Qiu, Félicien E. Ntone, Julius Y. Fonsah, Dora M. Njamnshi, Callixte T. Kuate, Roland F. Doh, Anne M. Kengne, Claude T. Tagny, Emilienne Nchindap, Léopoldine Kenmogne, Dora Mbanya, Mariana Cherner, Robert K. Heaton, Alfred K. Njamnshi

**Affiliations:** 1 Department of Pharmacology and Experimental Neuroscience, College of Medicine, University of Nebraska Medical Center, Omaha, Nebraska, United States of America; 2 Department of Biostatistics, College of Public Health, University of Nebraska Medical Center, Omaha, Nebraska, United States of America; 3 Faculty of Medicine and Biomedical Sciences, University of Yaoundé I, Yaoundé, Cameroon; 4 Department of Neurology, Yaoundé Central Hospital, Yaoundé, Cameroon; 5 HIV-Day Care Service, Yaoundé Central Hospital, Yaoundé, Cameroon; 6 Yaoundé University Teaching Hospital, Yaoundé, Cameroon; 7 HIV Neurobehavioral Research Center, School of Medicine, University of California San Diego, San Diego, California, United States of America; 8 Department of Psychiatry, School of Medicine, University of California San Diego, La Jolla, San Diego, California, United States of America; The University of New South Wales, Neuroscience Research Australia, AUSTRALIA

## Abstract

Depression is a leading cause of HIV/AIDS disease burden; it worsens health outcomes and quality of life. Addressing this problem requires accurate quantification of the extra burden of depression to HIV/AIDS in a given population, and knowledge of the baseline depression prevalence in the general population. There has been no previous study of depression in the general Cameroonian population. The current study attempts to address that important need. We used the Beck Depression Inventory-II to assess the prevalence and severity of depressive symptoms in 270 HIV-infected and seronegative Cameroonians. Univariate analyses showed a trend toward higher depressive symptoms among cases, compared to controls (p = 0.055), and among older subjects (>40 years), compared to younger subjects (≤40 years) (p = 0.059). Analysis of depression severity showed that 33.73% of cases had moderate-to-severe depressive symptoms, compared to 19.8% of controls (p<0.01). However, multivariable negative binomial regression analyses showed no effect of age, HIV status, CD4 levels, viral loads, ART, or opportunistic infections on the risk of depressive symptoms. Both univariate and multivariable regression analyses showed significantly higher risk of depressive symptoms among females compared to males; this was significant for both female controls and female cases. Female cases had significantly higher CD4 cell counts and lower viral loads, compared to males. Both univariate and multivariable regression analyses showed that lower education (≤10 years) was associated with increased risk of depressive symptoms. This study shows a high prevalence of depressive symptoms among seronegative controls and HIV-infected Cameroonians. Integrating care for mental disorders such as depression into primary health care and existing HIV/AIDS treatment programs in Cameroon may improve the wellbeing of the general population and could lower the HIV/AIDS burden.

## Introduction

Depression is a leading cause of disease burden. Recent studies showed that major depressive disorder is the 2^nd^ leading cause of disability worldwide [1, 2], and is a major cause of suicide [2–5]. By the year 2030, depression is projected to be one of the three leading causes of illness worldwide, together with HIV/AIDS and ischemic heart disease [1, 2]. Depression also contributes to the global burden of both non-communicable diseases such as ischemic heart disease [6], and communicable diseases such as HIV/AIDS [7–14]. Studies in both high- and low-income countries have reported an association between HIV/AIDS and depression, and demonstrated that depression is associated with worse HIV/AIDS outcomes and poorer quality of life [7–14].

The Beck Depression Inventory (BDI)-II is one of the most widely used tools in psychometric research (for review, see [15]). The BDI-II has been translated into many languages, validated in numerous studies, and shown to reliably measure the symptoms of depression in populations in both high-income [14–21] and resource-limited countries [7–13, 20, 22–24]. We have used and validated the BDI-II in Cameroon [25]. Previous studies have found high rates of depressive symptoms among HIV/AIDS patients in Cameroon, with reported prevalence of depression ranging from 21 to 63% [26–31]. However, all these studies only examined HIV-infected subjects and none included seronegative controls. There has been no study of depression in the general Cameroonian population, and the epidemiology of depression in Cameroon is not known. In the present study, using the BDI-II, we assessed the prevalence and severity of depression in HIV-infected and seronegative control individuals in Yaoundé, Cameroon. We further assessed the influence of age, gender and education on the occurrence of depression in both groups, as well as the effect of antiretroviral therapy, opportunistic infections, viral loads, and immune status on the risk of depression in infected individuals.

## 2. Materials and methods

### 2.1. Study design and ethical considerations

This cross-sectional study was part of an ongoing research project aimed at analyzing the influence of HIV genetic diversity on viral neuropathogenesis in Cameroon. The study was performed in accordance with guidelines of the Helsinki Declaration and was approved by the Cameroon National Ethics Committee, as well as the Institutional Review Board of the University of Nebraska Medical Center. Written informed consent was obtained from all the participants and data were processed using unique identifiers to ensure participant confidentiality.

### 2.2. Participants

A total of 270 HIV seropositive individuals and seronegative controls were consecutively recruited between 2008 and 2015. Cases were recruited from 1) the HIV voluntary counseling and testing section of the Day-care Service in the Yaoundé Central Hospital; 2) the Yaoundé Jamot Hospital; 3) the Efoulan District Hospital, Yaoundé; and 4) the Etoug-Ebe Baptist Health Center, Yaoundé. Controls were recruited from the same health services, as well as among 1) caregivers and visitors to the Neurology outpatient clinic and Day-care service in the Yaoundé Central Hospital; and 2) the Health and Social Welfare Centre of the University of Yaoundé-1. The purpose of the study and research procedures were fully explained to the participants, and adults at least 18 years old who gave a written consent were allowed to participate in the study. The exclusion criteria were: 1) present or past history of CNS disease unrelated to HIV, 2) head trauma, 3) current alcohol intoxication (blood alcohol content of each participant was measured using a Breathalyzer), 4) known psychiatric disease or treatment with antipsychotic drugs, and 5) ongoing systemic illness or fever (temperature of 37.5°C or higher). All subjects enrolled spoke French as their primary language of instruction and interviews were thus conducted in French.

### 2.3. Data collection

All participants provided demographic information, underwent a complete medical history, a general physical examination, and a thorough neurological assessment by neurologists at the Yaoundé Central Hospital to detect any focal neurological deficit suggestive of CNS opportunistic infection, before psychometric testing. This thorough clinical assessment of each subject combined with review of his or her prior medical history and subsequent laboratory data, ensured that potential confounding factors such as existing CNS opportunistic infections were ruled out. The Beck Depression Inventory-II [32] was administered to each subject by trained psychometrists in the Neuropsychology laboratory of the Neurology Department of the Yaoundé Central Hospital: a private, quiet and well-lit room. Psychometric testing was done prior to blood sample collection and laboratory analyses for HIV serology.

#### HIV serology, CD4 cell counts, and viral loads

Sample collection and all analyses were performed in the Hematology laboratory of the Yaoundé University Teaching Hospital, Cameroon. Venous blood samples were collected and stored at room temperature in the outpatient clinic and analyses performed in the Hematology laboratory within 6 hours of blood collection. The HIV status of each participant was determined using the rapid immunochromatographic HIV-1/2 test (Abbott Diagnostics, Chicago, IL, USA) and the Murex HIV antigen/antibody Combination ELISA (Abbott Diagnostics), according to the manufacturer’s instructions. A participant was considered HIV+ if he/she tested positive for the two tests, HIV- if negative for both tests, and discordant if positive for only one test. No discordant result was observed in this study.

Subjects’ CD4 T-lymphocyte counts were quantified by flow cytometry, using a Fluorescence Activated Cell Sorting (FACS) Count Instrumentation System and the BD FACSCount CD4 reagent kit (BD Biosciences, San Jose, CA, USA), according to the manufacturer’s instructions. The FACS instrument was calibrated and quality control tested before each experiment. For viral loads quantification, HIV RNA copy number in each plasma sample was quantified by reverse transcription polymerase chain reaction (RT-PCR), using Amplicor HIV-1 Monitor Test (Roche Diagnostic Systems, Pleasanton, CA), according to the manufacturer’s protocol. The assay detection limit was 50 viral copies / ml.

### 2.4. Psychometric instruments

#### 2.4.1. Beck depression inventory-II

The Beck Depression Inventory (BDI)-II [15, 32] is widely used to detect the presence of depressive mood and to measure the severity of depression. This instrument has been translated into many languages, including French, validated through back-translations and test-retest reliability. This validated French version of the BDI-II was shown to accurately diagnose depression in human studies in France [33, 34], Belgium [35], and Canada [36–38]. We previously adapted and validated this French version of the BDI-II in Cameroon [25], and in our current study, this psychometric test was used to detect depression and assess its severity. The BDI-II was administered to each subject by trained psychometrists in a face-to-face interview. The BDI-II consists of a 21-item questionnaire that assesses affective symptoms of depression, including hopelessness, irritability, feelings of guilt, pessimism, worthlessness, self-dislike, and suicidal thoughts; as well as somatic symptoms such as loss of appetite, fatigue, difficulties sleeping and concentrating. The answer to each question was scored on a scaled value of 0 to 3, and the total score determined the severity of depression: 0–13 indicating minimal depression; 14–19 indicating mild depression; 20–28 indicating moderate depression; 29–63 indicating severe depression [15, 32].

#### 2.4.2. Beck depression inventory fast-screen

The BDI- Fast Screen (FS) is a shorter 7-item instrument that includes questions extracted from the BDI-II [15, 32, 39]. The answer to each question was scored on a scaled value of 0 to 3, and the FS score determined the severity of depression: 0–3 indicating minimal depression; 4–8 indicating mild depression; 9–12 indicating moderate depression; 13–21 indicating severe depression [15, 32, 39].

### 2.5. Statistical analysis

The characteristics of participants were compared between HIV case and control groups using t-tests or Wilcoxon rank-sum tests for continuous variables, and the Chi-squared or Fisher’s exact tests for categorical variables. The outcomes of the study, Beck total and Beck FS scores, were compared between groups using the Wilcoxon rank-sum tests or Kruskal-Wallis tests because they were not normally distributed. Chi-squared tests, Fisher’s exact tests, or logistic regressions were used to determine the association between the severity of depression and the characteristics of participants. Multivariable negative binomial regressions were used to determine the effects of age, education, HIV status, gender, as well as the interaction between HIV status and gender on Beck total or Beck FS scores. Multivariable negative binomial regression analyses were also used to examine the effects of antiretroviral therapy (ART), CD4 levels, and viral loads on the Beck total or Beck FS scores among HIV-infected patients. Tukey-Kramer’s correction was used to control for all pairwise comparisons, and Dunnett’s correction was used for multiple comparisons with a control group [40]. All analyses were performed using SAS version 9 (SAS Institute, Inc., Cary, NC).

## 3. Results

### 3.1. Demographics

A total of 270 (169 HIV+ and 101 HIV-) adult Cameroonians were evaluated. Participants’ demographic characteristics are summarized in Table 1. Amongst the HIV+ subjects, 105 (62.9%) were on ART, whereas 64 (37.1%) were treatment naïve, and 43 (25.6%) had opportunistic infections and were on concurrent antibiotic treatment. Although cases and controls were recruited from the same health facilities and settings in Yaoundé, there were demographic differences between the two groups. Compared to cases, controls were significantly younger: female controls were younger than female cases, and male controls were younger than male cases (Table 1). Male cases were significantly older than female cases (P<0.01), but there was no significant difference between the ages of female controls and male controls. Compared to cases, controls were significantly more educated (Table 1). There was no significant difference in education level between female cases and male cases, or between female controls and male controls.

**Table 1:** Demographic characteristics of the HIV+ and HIV- groups.

	CASES (n = 169)	CONTROLS (n = 101)	P-value
**Cases on ART (n, %)**	105/167 (62.9)	-	
**Cases with OI (n, %)**	43/168 (25.6)	-	
**Age (mean ± SD)**	38.64 ± 9.11	29.47 ± 8.82	<0.0001
**Age > 40 years (n, %)**	66 (39.05)	13 (12.87)	<0.0001
**EDU (mean ± SD)**	9.79 ± 3.69	13.62 ± 3.62	<0.0001
**EDU ≤ 10 years (n, %)**	100 (59.17)	24 (23.76)	<0.0001
**EDU 11 to 13 years (n, %)**	43 (25.44)	20 (19.8)	
**EDU ≥ 14 years (n, %)**	26 (15.38)	57 (56.44)	
**Females (n, %)**	135 (79.88)	68 (67.33)	0.02
**Mean Female age ± SD (n)**	37.53 ± 8.82 (135)	29.87 ± 9.18(68)	<0.0001
**Mean Male age ± SD (n)**	43.03 ± 9.03(34)	28.64 ± 8.11(33)	<0.0001
**Mean Female EDU ± SD (n)**	9.49 ± 3.69(135)	13.5 ± 3.6 (68)	<0.0001
**Mean Male EDU ± SD (n)**	10.97 ± 3.48 (34)	13.88 ± 3.71(33)	0.002
**Speak French (%)**	169 (100)	101 (100)	0.99

F: females, M: male; EDU: education, ART: antiretroviral therapy; SD: standard deviation; n: sample size; OI: opportunistic infections

EDU ≤ 10 years corresponds to a maximum middle school (8^th^ grade) level education.

EDU 11 to 13 years corresponds to a high school (9^th^ to 12^th^ grade) level education.

EDU ≥ 14 years correspond to college and post-graduate level education.

### 3.2. Clinical and laboratory characteristics of cases

The clinical and laboratory characteristics of the 169 cases are summarized in Tables 2–4. The overall mean CD4 was 450.36 ± 261 cells/ μl; 464.31 ± 244.34 cells/ μl for females and 394.58 ± 317.78 cells/ μl for males; 367.8 ± 215 cells/ μl for treatment naïve cases and 503.5 ± 273 cells/ μl for cases on ART; 455.83 ± 242.7 cells/ μl for cases ≤ 40 years old and 442.17 ± 288.3 cells/ μl for cases > 40 years old. The overall median CD4 was 418 (range: 5 to 1657) cells/ μl, the median viral load was 50 (range: undetectable (≤ 50) to 14,964,000) copies/ml; and the median log viral load was 1.69 (range: 1.69 to 7.17) log copies/ml. The viral load was ≤ the 50 copies/ml detection limit in 67.6% of cases. Compared to male cases, female cases had significantly higher CD4 cell counts (Table 2, P = 0.04) and significantly lower viral loads (Table 2, P = 0.02). Compared to treatment naïve cases, subjects on ART had significantly higher CD4 cells (Table 3, P = 0.0009) and significantly lower viral loads (Table 3, P < 0.0001). There was no significant difference between younger and older cases with respect to CD4 counts or viral loads (Table 4).

**Table 2:** Clinical and laboratory characteristics of HIV-infected subjects: CD4 and viral loads of males and females cases.

Variables	Gender	N	Median	Minimum	Maximum	P-value
**CD4 (cells/ μl)**	F	132	449.5	5	1233	**0.04**
	M	33	295	34	1657	
**VL (copies/ ml)**	F	135	50	50	5,389,975	**0.02**
	M	34	50	50	14,964,000	
**VL (Log copies/ml)**	F	135	1.69	1.69	6.73	**0.02**
	M	34	1.69	1.69	7.17	

**Table 3:** Clinical and laboratory characteristics of HIV-infected subjects: CD4 and viral loads of cases on ART and treatment naïve.

Variables	ART	N	Median	Minimum	Maximum	P-value
**CD4 (cells/ μl)**	No	60	319.5	34	894	**0.0009**
	Yes	103	483	5	1657	
**VL (copies/ml)**	No	62	7460.5	50	14,964,000	**< .0001**
	Yes	105	50	50	1,380,311	
**VL (Log copies/ml)**	No	62	3.25	1.69	7.17	**< .0001**
	Yes	105	1.69	1.69	6.14	

**Table 4:** Clinical and laboratory characteristics of HIV-infected subjects: CD4 and viral loads of younger and older cases.

Variables	Age	N	Median	Minimum	Maximum	P-value
**CD4 (cells/ μl)**	< = 40	99	421	5	1233	0.54
	>40	66	395	70	1657	
**VL (copies/ml)**	< = 40	103	50	50	5,246,000	0.29
	>40	66	50	50	14,964,000	
**VL (Log copies/ml)**	< = 40	103	1.69	1.69	6.71	0.3
	>40	66	1.69	1.69	7.17	

N: sample size; VL: viral loads; ART: antiretroviral therapy; F: females, M: males

### 3.3. Effects of HIV status on depressive symptoms among Cameroonians

Univariate analysis showed marginally higher median Beck total scores (P = 0.05) among cases compared to controls (Table 5). Further multivariable negative binomial regression analyses showed no significant difference in Beck total scores (Table 6, P = 0.91) or Beck FS scores (Table 7, P = 0.13) of cases and controls; and showed no interaction between gender and HIV status for both Beck total scores (Table 6, P = 0.88) and Beck FS scores (Table 7, P = 0.18). Analysis of depression severity based on Beck total scores showed mild-to-no depression in 66.27% of cases, and 80% of controls, and moderate-to-severe depression in 33.73% of cases compared to 19.8% among controls (Table 8, P = 0.01). However, the difference in Beck FS scores between cases and controls was not significant by univariate analysis.

**Table 5:** Univariate analysis of depression risks (BDI-II) among Cameroonian subjects.

Outcome	Variables		N	Median Score	Range	P-value
**BECK Total Score**	Status	Case	169	14	0–60	**0.055**
		Control	101	12	0–45	
	Age (y)	≤ 40	191	12	0–60	**0.059**
		> 40	79	16	0–44	
	EDU (y)	≥ 14	83	9	0–42	**0.002**
		≤ 10	124	14.5	0–44	
		11 to 13	63	14	0–60	
	Gender	F	203	14	0–60	**0.02**
		M	67	10	0–40	
**BECK FS Score**	Status	Case	168	4	0–18	0.13
		Control	101	3	0–13	
	Age (y)	≤ 40	191	3	0–18	0.75
		> 40	78	3	0–14	
	EDU (y)	≥ 14	82	2	0–17	**0.0004**
		≤ 10	124	3	0–14	
		11 to 13	63	5	0–18	
	Gender	F	202	4	0–18	**0.04**
		M	67	3	0–10	

EDU: education; y: years; F: females, M: males; cases: human immunodeficiency virus (HIV)+; controls: HIV-; ART: antiretroviral therapy; OI: opportunistic infections; N: sample size

**Table 6:** Negative binomial regression analysis of depression risks (BDI-II) among Cameroonian subjects: Analysis based on BECK Total scores.

Variables		Coefficient	95% CI		P-value
Age (years)	> 40	0.19	-0.02	0.41	**0.08**
	≤ 40	Reference			
EDU ≥ 14 years ^§^		-0.25	-0.52	-0.02	**0.07**
EDU 11 to 13 years ^§^		0.13	-0.14	0.40	0.46
EDU ≤ 10 years ^§^		Reference			
Status	Cases	-0.02	-0.42	0.37	0.91
	Controls	Reference			
Gender	F	0.28	-0.04	0.60	**0.09**
	M	Reference			
Gender*Status	F cases	0.03	-0.40	0.47	0.88
	M controls	Reference			

F: female; M: male; CI: confidence interval;

^§^ Dunnett-Hsu’s method used to control for multiple comparisons.

**Table 7:** Negative binomial regression analysis of depression risks (BDI-II) among Cameroonian subjects: Analysis based on Beck FS scores.

Variables		Coefficient	95% CI		P-value
Age (years)	> 40	0.02	-0.23	0.28	0.85
	≤ 40	Reference			
EDU ≥ 14 years^§^	≥ 14	-0.20	-0.52	0.11	0.26
EDU 11 to 13 years ^§^	10 to 13	0.31	0.01	0.61	**0.04**
EDU ≤ 10 years ^§^	≤ 10	Reference			
Gender	F	0.53	0.14	0.92	**0.008**
	M	Reference			
Status	Cases	0.36	-0.11	0.82	0.13
	Controls	Reference			
Gender*Status	F Cases	-0.35	-0.87	0.17	0.18
	M Controls	Reference			

F: female; M: male; CI: confidence interval; FS: fast screen;

^§^ Dunnett-Hsu’s method used to control for multiple comparisons.

**Table 8:** Severity of depression among cases and controls.

**Status**	**Variables**		**Cases (n = 169)**	**Controls (n = 101)**	**P-value**
**ALL SUBJECTS**	**BECK Total Score**	Minimal/Mild, n (%)	112 (66.27)	81 (80.2)	**0.01**
		Moderate/Severe, n (%)	57 (33.73)	20 (19.8)	
	**BECK FS Score**	Minimal/Mild, n (%)	143 (85.12)	90 (89.11)	0.35
		Moderate/Severe, n (%)	25 (14.88)	11 (10.89)	
**Status**	**Variables**		**NoART (n = 62)**	**ART (n = 105)**	**P-value**
**CASES**	**BECK Total Score**	Minimal/Mild, n (%)	37 (59.68)	73 (69.52)	0.19
		Moderate/Severe, n (%)	25 (40.32)	32 (30.48)	
	**BECK FS Score**	Minimal/Mild, n (%)	51 (82.26)	90 (85.71)	0.55
		Moderate/Severe, n (%)	11 (17.74)	15 (14.29)	
**Status**	**Variables**		**F (n = 135)**	**M (n = 34)**	**P-value**
**CASES**	**BECK Total Score**	Minimal/Mild, n (%)	88 (65.19)	24 (70.59)	0.55
		Moderate/Severe, n (%)	47 (34.81)	10 (29.41)	
	**BECK FS Score**	Minimal/Mild, n (%)	111 (82.22)	32 (94.12)	**0.09**
		Moderate/Severe, n (%)	24 (17.78)	2 (5.88)	
**Status**	**Variables**		**Age < = 40 (n = 103)**	**Age >40 (n = 66)**	**P-value**
**CASES**	**BECK Total Score**	Minimal/Mild, n (%)	69 (66.99)	43 (65.15)	0.81
		Moderate/Severe, n (%)	34 (33.01)	23 (34.85)	
	**BECK FS Score**	Minimal/Mild, n (%)	87 (84.47)	56 (84.85)	0.95
		Moderate/Severe, n (%)	16 (15.53)	10 (15.15)	
**Status**	**Variables**		**F (n = 68)**	**M (n = 33)**	**P-value**
**CONTROLS**	**BECK Total Score**	Minimal/Mild, n (%)	54 (79.41)	27 (81.82)	0.78
		Moderate/Severe, n (%)	14 (20.59)	6 (18.18)	
	**BECK FS Score**	Minimal/Mild, n (%)	59 (86.76)	31 (93.94)	0.5
		Moderate/Severe, n (%)	9 (13.24)	2 (6.06)	
**Status**	**Variables**		**Age < = 40 (n = 103)**	**Age >40 (n = 66)**	**P-value**
**CONTROLS**	**BECK Total Score**	Minimal/Mild, n (%)	73 (82.95)	8 (61.54)	0.13
		Moderate/Severe, n (%)	15 (17.05)	5 (38.46)	
	**BECK FS Score**	Minimal/Mild, n (%)	80 (90.91)	10 (76.92)	0.15
		Moderate/Severe, n (%)	8 (9.09)	3 (23.08)	

N: sample size

Further univariate analysis of cases showed that CD4 levels and viral loads had no effect on Beck total scores or Beck FS scores (S1 Table). Multivariable negative binomial regression analyses of cases confirmed these results and showed no significant difference in Beck total scores (S2 Table) or Beck FS scores (S3 Table) of cases with lower CD4 cells and cases with higher CD4 cells. Analysis of depression severity (cases with minimal/mild depression vs. cases with moderate/severe depression) based on Beck total scores and Beck FS scores showed no significant effect of CD4 levels or viral loads on depression. Similar results were obtained using logistic regression analysis (S4 and S5 Tables), Chi-squared tests (S6 and S7 Tables), and Wilcoxon-rank sum tests (data not shown).

### 3.4. Effects of age on depressive symptoms among Cameroonians

Univariate analysis of all subjects (all cases and controls combined) based on age showed a trend toward higher median Beck total scores among subjects over 40 years old, compared to younger subjects (P = 0.059, Table 5). Separate univariate analyses of controls and cases showed no significant difference in Beck total scores and Beck FS scores between older and younger controls, and no significant difference in Beck total scores or Beck FS scores between older and younger cases. Quantification of depression severity using Beck total scores or Beck FS scores also showed no significant differences in the proportion of older and younger cases or older and younger controls with moderate-to-severe depression (Table 8). However, analysis of controls’ Beck total scores showed that a higher proportion of older controls (38.46%) had moderate-to-severe depression, compared to younger controls (only 17%). Analyses based on Beck FS scores also showed that 23% of older controls had moderate-to-severe depression, compared to only 9% of younger controls, but these differences were not statistically significant (Table 8).

Multivariable negative binomial regression analyses showed marginally higher mean log Beck total scores in subjects over 40 years old, compared to younger subjects, although the difference did not reach statistical significance (P = 0.08, Table 6). Univariate and multivariable analyses of Beck FS scores showed no difference between the older and younger groups (Tables 5 and 7). Further multivariable analysis of cases, using negative binomial regression analysis, showed no significant difference in Beck total scores (S2 Table) or Beck FS scores (S3 Table) of older and younger cases. Multivariable analysis of depression severity based on Beck total scores (S4 Table) and Beck FS scores (S5 Table) also showed no effect of age on the severity of depression among cases.

### 3.5. Effects of education on depressive symptoms among Cameroonians

In univariate analysis of all subjects (cases and controls combined), subjects with fewer than 14 years of education had significantly higher median Beck total scores (Table 5, P = 0.002) and Beck FS scores (Table 5, P = 0.0004), compared to subjects who attended college (≥14 years of education). Separate univariate analyses of controls and cases showed that compared to less educated controls, those with at least 14 years of schooling had significantly lower Beck total scores (P = 0.04) and lower Beck FS scores (P<0.0005). However, analysis of cases showed no significant differences in Beck total scores or Beck FS scores, between the small numbers of cases with high levels of education, as compared to a much larger subgroup with lower levels of education.

Multivariable negative binomial regression analyses of all subjects showed lower mean log Beck scores in subjects with at least 14 years of education, compared to less educated subjects (Table 6, P = 0.07), but no significant difference was observed between subjects with a maximum 10 years education and those with 11 to 13 years of education (Table 6, P = 0.46). Multivariable negative binomial regression analysis of Beck FS scores showed no significant differences between subjects with ≥14 years of education, compared to less educated subjects, but showed significant differences between subjects with a maximum 10 years education and those with 11 to 13 years of education (Table 7, P = 0.04).

Additional multivariable negative binomial regression analysis of cases showed no significant difference in Beck total scores (S2 Table) or Beck FS scores (S3 Table) of cases with ≥14 years of education, compared to less educated cases. Multivariable logistic regression analyses of depression severity (cases with minimal/mild vs. moderate/severe depression) based on Beck total scores showed no effect of education on the severity of depression among cases (S4 Table). However, analysis of depression severity based on Beck FS scores showed higher scores in subjects with 11 to 13 years of education compared to those with a maximum 10 years education (S5 Table, P = 0.04).

### 3.6. Effects of gender on depressive symptoms among Cameroonians

Univariate analysis of all subjects (cases and controls combined) showed significantly higher median Beck total scores (Table 5, P = 0.02) and Beck FS scores (Table 5, P = 0.04) among female compared to male participants. Separate univariate analyses of controls and cases showed higher Beck total scores (P = 0.08) and significantly higher Beck FS scores (P = 0.009) among female controls compared to male controls. Analysis of cases showed mean gender differences of the same magnitude (albeit not significant) in Beck total scores. Quantification of depression severity using Beck total scores showed no significant differences in the proportion of males and females with minimal-to-mild, and moderate-to-severe depression. However, Chi-squared analysis of Beck FS scores showed a somewhat higher proportion of female cases (17.8%) with moderate-to-severe depression, compared to male cases (5.88%) (Table 8, P<0.09). Similarly, a higher proportion of female controls (13.24%) had moderate-to-severe depression, compared to male controls (6%), although the difference was not statistically significant (Table 8).

Multivariable negative binomial regression analyses showed that there was no significant interaction between gender and status for each outcome (Table 6, P = 0.88 and Table 7 P = 0.18). Male controls had lower mean log Beck FS score than female cases (P = 0.03) and lower mean log Beck FS score than female controls (P = 0.04). Additional multivariable regression analysis without the interaction term showed significantly higher mean log Beck total scores (Table 9, P = 0.008) and higher mean log Beck FS scores (Table 9, P = 0.01) among female compared to male participants.

**Table 9:** Negative binomial regression analysis of depression risks (BDI-II) among Cameroonian subjects: Analysis after withdrawing the interaction term (gender*status).

Outcome	Gender	Estimate	95% CI		P-value
Beck Total scores	F	0.30	0.08	0.52	**0.008**
	M	Reference			
Beck FS scores	F	0.33	0.07	0.59	**0.01**
	M	Reference			

F: female; M: male; CI: confidence interval; FS: fast screen

Further multivariable negative binomial repression analysis of cases showed significantly higher mean log Beck total scores among female cases, compared to male cases (S2 Table, P = 0.02), but these difference were not significant for Beck FS scores (S3 Table). Multivariable logistic regression analysis of depression severity (cases with minimal-to-mild vs. moderate-to-severe depression) showed increased depression among female cases compared to male cases (coefficient: 0.77, 95% confidence interval: -0.19 to 1.73), although this difference did not reach statistical significance (S4 Table). Analysis of depression severity based on Beck FS scores showed significantly increased depression among female cases compared to male cases (coefficient: 1.73, 95% confidence interval: 0.10 to 3.36; P = 0.04) (S5 Table).

### 3.7. Effects of ART use and opportunistic infections on depressive symptoms among HIV-infected Cameroonians

Univariate analysis of cases showed that ART use or the presence of opportunistic infections did not significantly change the Beck total or FS scores, compared to ART-naïve or cases with no opportunistic infections. Quantification of depression severity among cases using Chi-Squared tests, and both Beck total and FS scores, showed no significant differences in the proportion of treatment-naïve cases and cases on ART with moderate-to-severe depression (Table 8). Similarly, logistic regression analysis of depression severity, using both Beck total scores (S4 Table) and FS scores (S5 Table) showed no significant effect of ART use on the risk of depression among cases. Multivariable negative binomial regression analyses confirmed univariate analysis results and showed no significant difference in mean log Beck total or FS scores of cases on ART, treatment-naïve cases, and uninfected controls. Further multivariable negative binomial regression analyses of cases showed no significant difference in mean log Beck total or FS scores of cases on ART and treatment-naïve cases (S2 and S3 Tables).

## Discussion

In this study, we analyzed the prevalence and severity of depressive symptoms in both uninfected controls and HIV-infected subjects in Cameroon. Our data show relatively high rates of depressive symptoms in uninfected control subjects in Cameroon; about 20% of seronegative controls had moderate-to-severe depression, including older and younger subjects, female (20.59%) and male (18%) controls. This prevalence is within the range of depression prevalence reported in the general population in other African countries, including studies using different psychometric instruments such as the Center for Epidemiologic Studies Depression Scale (CES-D) in South Africa that showed 20.5% and 13.5% prevalence of depressive symptoms among females and males, respectively [41]. A Ugandan study using the Hopkins Symptom Checklist-25, showed a 29% depression prevalence [42]. The 20% prevalence of depressive symptoms in our seronegative controls is also in line with findings in other countries using the BDI-II instrument. A systematic review of 54 studies on depression, including 31 cross-sectional studies involving 9447 individuals, and 23 longitudinal studies involving 8113 individuals, showed that the pooled prevalence of depressive symptoms was 28.8% [20]. For the 9 studies in this review that used BDI-II, the sample size varied from 43 to 268 subjects, and four studies showed a 10 to 25.6% prevalence of mild-to-severe depressive symptoms [43–46] while five studies showed 30 to 63% prevalence of mild-to-severe depressive symptoms [45, 47–50]. However, none of these 9 studies that used BDI-II was conducted in Africa; only one of the total 54 studies included in this review was conducted in Africa (Kenya) and only two were conducted in low-income countries.

Other population studies in SSA and the US reported lower depression prevalence. Studies of the general population and seronegative adults in Nigeria [13, 51], Ghana [52], and South Africa [52–54] using the Composite International Diagnostic Interview (CIDI) or BDI-II [13] showed 2.7% to 12% depression rates. Studies of US adult population, including African American [55], using the PHQ-9 [56] or BDI-II [55, 57], showed 6% to 11% prevalence of moderate-to-severe depression. These prevalence estimates are lower than the 20% rate in our controls, and depression rates reported in other studies [41, 42, 45, 47–50]. It is possible that the different psychometric instruments used in these studies may account for some of the differences. It is also possible that the standard cut-off scores of these psychometric instruments, most of which were standardized and established based on the US population, may not be optimal when applied to populations in other countries.

Factors such as unemployment and poverty have been associated with increased risk of depressed mood [58–62]. Cameroon has a 33.6% unemployment rate, 75% underemployment rate, and 44.6% of Cameroon’s 22.8 million inhabitants live near poverty or in poverty [63]. These factors, combined with other societal factors associated with poverty and hardship, could help explain the high prevalence of depressive symptoms in apparently healthy individuals in our study. This shows that depression may be a serious health issue in the general Cameroon population, and treatment and care for depression and other mental disorders should be included in the country’s health care system.

In the current study, univariate analyses of Beck total and FS scores showed significantly higher prevalence of depressive symptoms among HIV-infected subjects in Cameroon, with 33.73% having moderate-to-severe depression, compared to 20% of controls. Previous studies using different psychometric instruments also showed the presence of depressive symptoms among HIV-infected subjects in Cameroon. Studies using the PHQ-9 [26, 27] or CIDI [28], with a sample size of 100 to 400 subjects, reported a 63% [26], 28.7% [27], and 21% [28] prevalence of moderate-to-severe depression among HIV-infected subjects. Our findings of a 33.7% prevalence of moderate-to-severe depression among HIV-infected subjects agree with these previous findings and confirm that depression is a serious health issue among HIV-infected, as well as uninfected individuals in Cameroon. Furthermore, our results are in agreement with studies reported elsewhere using the BDI-II. Studies of HIV/AIDS patients in Brazil showed moderate-to-severe depression in 27.6 to 36% of patients, and showed that the presence of depressive symptoms was associated with lower quality of life [7–9]. A study of HIV-infected subjects in Korea [14] and France [62] showed moderate-to-severe depressive symptoms in 19.7 to 21% of the subjects. Studies in other SSA countries using BDI-II also showed increased prevalence of moderate-to-severe depressive symptoms among HIV-infected subjects, including in 18.9% of HIV-infected adolescents in Malawi [10], in 37.6 to 40% of infected subjects in South Africa [11, 12] and in 29 to 32% of HIV/AIDS patients in Nigeria [13].

Although univariate analyses showed higher Beck total scores among cases compared to controls in our current study, there was no significant difference in Beck FS scores, and multivariable analysis controlling for other co-factors such as age, education, and gender showed no significant difference in Beck total or FS scores of cases and controls. This suggests that these cofactors may have significant effects on the differential results observed. Further analyses showed that age did not significantly influence the risk of depressive symptoms either among cases or among controls, but education did. Both univariate and multivariable analyses showed that for the control group, having a lower education level was associated with increased risk of depressive symptoms, compared to those with college or postgraduate education. Although education did not significantly impact the prevalence of depressive symptoms among cases, analysis of Beck FS scores showed impact of education on depression severity. Our data confirmed previous findings in other countries that lower education level is associated with increased risk of depression. Studies in Brazil [9, 24], Ghana [61], and France [62] showed that depressive symptoms among HIV-infected subjects were associated with lower education, low income, unemployment and lower quality of life. However, we did not have information on the employment status and income levels of our study population, and could not assess their impact on the risk of depression.

Studies in both high-income and resource-limited countries have shown that depression is more common among women than men [13, 16, 64–67] and WHO estimates that the burden of depression among women is 50% higher than in men [68, 69]. In the current study both univariate and multivariable analyses showed significantly higher prevalence of depressive symptoms among females, compared to males, and subgroup multivariable analyses showed gender differences of the same magnitude. Analysis of depression severity also indicated that the percentage of female cases with moderate-to-severe depression was triple that of male cases, and the percentage of female controls with moderate-to-severe depression was double that of male controls. These data confirmed reports from other countries regarding gender differences in rates and severity of depressive symptoms. Our study showed that CD4 levels, viral loads, ART use, or opportunistic infections were not associated to the likelihood of having depression among cases. This agrees with previous studies of HIV/AIDS patients in both urban [26] and rural [27] areas in Cameroon that showed, using PHQ-9, a lack of correlation between ART use and depression.

In summary, our current study shows high rates of depressive symptoms consistent with moderate-to-severe depression in both seronegative controls (20%) and HIV-infected (33.7%) Cameroonians, suggesting that it is not only necessary to integrate care for mental disorders such as depression into existing HIV/AIDS care programs in Cameroon, but also into the general primary care system, as this would likely reduce the morbidity and mortality from other diseases in the country.

## Study Limitations

Like most psychometric tests currently used to detect and characterize depression, the BDI-II is a self-report inventory, and scores can be influenced by subjective biases in filling out the forms, as well as by the way questionnaires are administered, and by whom [39]. To minimize these limitations, all questionnaires were administered to each subject by trained psychometrists in the Neuropsychology laboratory, a private, quiet and well-lit room in the Neurology Department of Yaoundé Central Hospital. For each subject, the same psychometrists who conducted the interview also scored each form, and our findings are within the range of depression prevalence reported among the general population [20, 41–50] and HIV-infected subjects [7–9, 11–13] in other studies. Another limitation of this study is the fact that compared to cases, controls were much younger, more educated, and had a higher proportion of male participants. We attempted to address this limitation by performing separate within group analyses, looking at within-group effects of age, education, and gender on BDI-II scores. Additional limitations include the absence of employment and income status of study participants; factors such as unemployment, low income and poverty are associated with increased stress and higher risk of depression [13, 58–62, 67]. This study was conducted in Yaoundé, a large urban area, using a convenience sample of individuals whose characteristics might not be representative of Cameroon’s general population. Follow-up studies with larger sample size using subjects from other areas of Cameroon, including semi-urban and rural areas, will be required to generalize the findings, and to better understand the burden and determinants of depressive disorders in Cameroon.

## Supporting information

S1 Table Univariate analysis of the effect of CD4 count and viral loads on depression risks (BDI-II) among HIV-infected Cameroonians. (DOCX)

S2 Table Negative binomial regression analysis of depression risks (BDI-II) among HIV-infected Cameroonians: Analysis based on BECK total scores. (DOCX)

S3 Table Negative binomial regression analysis of depression risks (BDI-II) among HIV-infected Cameroonians: Analysis based on Beck FS scores. (DOCX)

S4 Table Logistic regression analysis of depression severity among HIV-infected Cameroonians: Analysis using the binary depression outcome (Minimal/Mild vs. Moderate/Severe) based on BECK total scores among cases. (DOCX)

S5 Table Logistic regression analysis of depression severity among HIV-infected Cameroonians: Analysis using the binary depression outcome (Minimal/Mild vs. Moderate/Severe) based on BECK FS score among cases. (DOCX)

S6 Table Severity of depression among HIV-infected Cameroonians: Analysis based on CD4 cell counts. (DOCX)

S7 Table Severity of depression among HIV-infected Cameroonians: Analysis based on viral loads. (DOCX)
