# Supplementary material for: Depressive symptoms in HIV-infected and seronegative control subjects in Cameroon: Effect of age, education and gender
Source: PLoS One. 2017 Feb 23;12(2):e0171956. doi: 10.1371/journal.pone.0171956 (PMC5322951; doi:10.1371/journal.pone.0171956)
Supplement: S1 Table — (DOCX) [file pone.0171956.s001.docx]

**S1 Table. Univariate analysis of the effect of CD4 count and viral loads on depression risks (BDI-II) among HIV-infected Cameroonians.**

| **Outcome** | **Variables** |  | **N** | **MedianScore** | **Range** | **P-value** |
| --- | --- | --- | --- | --- | --- | --- |
| **BECK Total Score** | CD4 (cells /µl) | CD4 ≤ 200 | 27 | 13 | 0-43 | 0.61 |
|  |  | CD4 > 200 | 138 | 14.5 | 0-60 |  |
|  | CD4 (cells /µl) | CD4 < 350 | 64 | 14 | 0-43 | 0.92 |
|  |  | CD4 ≥ 350 | 101 | 14 | 0-60 |  |
|  | CD4 (cells /µl) | CD4 ≥ 500 | 63 | 14 | 0-60 | 0.99 |
|  |  | CD4 < 500 | 102 | 14 | 0-46 |  |
|  | Viral load (copies / ml) | VL ≥ 100,000 | 19 | 12 | 3-42 | 0.99 |
|  |  | 50 > VL <100,000 | 36 | 13 | 0-43 |  |
|  |  | VL < 50 | 114 | 15 | 0-60 |  |
|  | Viral load (copies/ml) | VL ≥ 50 | 55 | 13 | 0-43 | 0.99 |
|  |  | VL < 50 | 114 | 15 | 0-60 |  |
| **BECK FS Score** | CD4 (cells /µl) | CD4 ≤ 200 | 26 | 3 | 0-14 | 0.71 |
|  |  | CD4 > 200 | 138 | 4 | 0-18 |  |
|  | CD4 (cells /µl) | CD4 < 350 | 63 | 4 | 0-14 | 0.74 |
|  |  | CD4 ≥ 350 | 101 | 3 | 0-18 |  |
|  | CD4 (cells /µl) | CD4 ≥ 500 | 63 | 4 | 0-18 | 0.56 |
|  |  | CD4 < 500 | 101 | 3 | 0-17 |  |
|  | Viral load (copies/ml) | VL ≥ 100,000 | 19 | 4 | 0-14 | 0.9 |
|  |  | 50 > VL <100,000 | 36 | 3 | 0-17 |  |
|  |  | VL < 50 | 113 | 4 | 0-18 |  |
|  | Viral load (copies/ml) | VL ≥ 50 | 55 | 3 | 0-17 | 0.69 |
|  |  | VL < 50 | 113 | 4 | 0-18 |  |

N: sample size; VL: viral loads
