# Supplementary material for: Depressive symptoms in HIV-infected and seronegative control subjects in Cameroon: Effect of age, education and gender
Source: PLoS One. 2017 Feb 23;12(2):e0171956. doi: 10.1371/journal.pone.0171956 (PMC5322951; doi:10.1371/journal.pone.0171956)
Supplement: S3 Table — (DOCX) [file pone.0171956.s003.docx]

**S3 Table. Negative binomial regression analysis of depression risks (BDI-II) among HIV-infected Cameroonians: Analysis based on Beck FS scores.**

| **Variables** | | **Coefficient** | **95% CI** | | **P-value** |
| --- | --- | --- | --- | --- | --- |
| Age (years) | > 40 | 0.03 | -0.28 | 0.34 | 0.84 |
|  | ≤ 40 | Reference | |  |  |
| EDU ≥ 14 years ^§^ |  | 0.05 | -0.43 | 0.52 | 0.97 |
| EDU 11 to 13 years ^§^ |  | 0.29 | -0.07 | 0.66 | 0.14 |
| EDU ≤ 10 years ^§^ |  | Reference | |  |  |
| Gender | F | 0.24 | -0.14 | 0.61 | 0.22 |
|  | M | Reference | |  |  |
| ART | Yes | -0.21 | -0.51 | 0.09 | 0.17 |
|  | No | Reference | |  |  |
| CD4 | CD4 < 500 cells /µl | -0.03 | -0.31 | 0.26 | 0.86 |
|  | CD4 ≥ 500 cells /µl | Reference |  |  |  |

EDU: education; ART: antiretroviral therapy; F: female; M: male; CI: confidence interval; FS: fast screen. ^§^ Dunnett-Hsu’s method used to control for multiple comparisons.
