# Supplementary material for: Depressive symptoms in HIV-infected and seronegative control subjects in Cameroon: Effect of age, education and gender
Source: PLoS One. 2017 Feb 23;12(2):e0171956. doi: 10.1371/journal.pone.0171956 (PMC5322951; doi:10.1371/journal.pone.0171956)
Supplement: S5 Table — (DOCX) [file pone.0171956.s005.docx]

**S5 Table. Logistic regression analysis of depression severity among HIV-infected Cameroonians: Analysis using the binary depression outcome (Minimal/Mild vs. Moderate/Severe) based on BECK FS score among cases.**

| **Variables** | | **Coefficient** | **95% CI** | | **P-value** |
| --- | --- | --- | --- | --- | --- |
| Age (years) | > 40 | 0.32 | -0.66 | 1.30 | 0.52 |
|  | ≤ 40 | Reference | |  |  |
| EDU ≥ 14 years ^§^ |  | 0.27 | -1.34 | 1.88 | 0.91 |
| EDU 11 to 13 years ^§^ |  | 1.18 | 0.03 | 2.33 | **0.04** |
| EDU ≤ 10 years ^§^ |  | Reference | |  |  |
| Gender | F | 1.73 | 0.10 | 3.36 | **0.04** |
|  | M | Reference | |  |  |
| ART | Yes | -0.63 | -1.62 | 0.36 | 0.21 |
|  | No | Reference | |  |  |
| CD4 counts | CD4 < 500 cells /µl | 0.16 | -0.79 | 1.11 | 0.74 |
|  | CD4 ≥ 500 cells /µl | Reference |  |  |  |

EDU: education; ART: antiretroviral therapy; F: female; M: male; CI: confidence interval; FS: fast screen. ^§^ Dunnett-Hsu’s method used to control for multiple comparisons.
