# Supplementary material for: Depressive symptoms in HIV-infected and seronegative control subjects in Cameroon: Effect of age, education and gender
Source: PLoS One. 2017 Feb 23;12(2):e0171956. doi: 10.1371/journal.pone.0171956 (PMC5322951; doi:10.1371/journal.pone.0171956)
Supplement: S6 Table — (DOCX) [file pone.0171956.s006.docx]

**S6 Table: Severity of depression among HIV-infected Cameroonians: Analysis based on CD4 cell counts**

|  | **Variables** | **CD4 ≥ 500 cells /µl (n=63)** | **CD4 < 500 cells /µl (n=102)** | **P-value** |
| --- | --- | --- | --- | --- |
| **BECK Total Score** | Minimal/Mild, n (%) | 42 (66.67) | 68 (66.67) | 0.99 |
|  | Moderate/Severe, n (%) | 21 (33.33) | 34 (33.33) |  |
| **BECK FS Score** | Minimal/Mild, n (%) | 54 (85.71) | 86 (85.15) | 0.92 |
|  | Moderate/Severe, n (%) | 9 (14.29) | 15 (14.85) |  |

N: sample size
