# Supplementary material for: Depressive symptoms in HIV-infected and seronegative control subjects in Cameroon: Effect of age, education and gender
Source: PLoS One. 2017 Feb 23;12(2):e0171956. doi: 10.1371/journal.pone.0171956 (PMC5322951; doi:10.1371/journal.pone.0171956)
Supplement: S7 Table — (DOCX) [file pone.0171956.s007.docx]

**S7 Table. Severity of depression among HIV-infected Cameroonians: Analysis based on viral loads**

|  | **Variables** | **VL detectable (≥ 50 copies/ml) (n=55)** | **VL non-detectable (< 50 copies/ml) (n=114)** | **P-value** |
| --- | --- | --- | --- | --- |
| **BECK Total Score** | Minimal/Mild, n (%) | 34 (61.82) | 78 (68.42) | 0.4 |
|  | Moderate/Severe, n (%) | 21 (38.18) | 36 (31.58) |  |
| **BECK FS Score** | Minimal/Mild, n (%) | 47 (85.45) | 96 (84.96) | 0.93 |
|  | Moderate/Severe, n (%) | 8 (14.55) | 17 (15.04) |  |

N: sample size; VL: viral load.
